# Atlanta Medical College

**Published:** 1879-02

**Authors:** 


### ATLANTA MEDICAL COLLEGE.

This institution has now nearly passed through its twenty-second session. The number of students in attendance the present course is over one hundred and thirty, as is shown by the *bona-fide* matriculates on the Dean's book. Of this number, thirty-five to forty have made application for the degree. The class, one of the most attentive and orderly we have seen, bids fair to afford some valuable practitioners.

The commencement exercises will take place on Tuesday night, the fourth of March, before a public audience, in DeGive's Opera House.

The term, which for a number of years consisted of barely four months, has been lengthened to a period of more than four and a half months. Commencing the fifteenth of November, the course does not conclude till the fourth of the following March. The half month in November is not, as is sometimes found, a preparatory, introductory or catch-student course of two weeks, but is a *bona-fide* part of the regular term, and published in the announcement as such.

This addition to the length of the course of lectures is necessary to the more perfect and complete teaching in each branch, and still further lengthening would not be amiss in several departments.

